# Global trends and development of acupuncture for stroke: A review and bibliometric analysis

**DOI:** 10.1097/MD.0000000000036984

**Published:** 2024-01-19

**Authors:** Chang-Jiang Cheng, Hai-Bo Yu

**Affiliations:** a The Fourth Clinical Medical College of Guangzhou University of Chinese Medicine, Shenzhen, China; b Shenzhen Traditional Chinese Medicine Hospital, Shenzhen, China.

**Keywords:** acupuncture, bibliometric analysis, rehabilitation, stroke

## Abstract

The objective of this review is to elaborate on the status, hotspots, and trends of researches on acupuncture for stroke over the past 26 years. Publications about acupuncture for stroke were downloaded from the Web of Science Core Collection, and these papers were published up to December 31, 2022. A bibliometric analysis of acupuncture for stroke was conducted by CiteSpace (6.2.R4) and VOSviewer (1.6.17). In this study, VOSviewer was used for visual analysis of countries, institutions, authors, journals, keywords, and co-cited references. CiteSpace was used to draw a keyword burst map and a co-cited reference burst map. A total of 534 papers were obtained from the Web of Science Core Collection. The number of papers per year showed a rapid upward trend. The most productive country and institution in this field were China (452) and the Fujian University of Traditional Chinese Medicine (43), respectively. Tao Jing had the highest number of articles (34), and EZ Longa was the most popular author (129 co-citations). *Neural Regeneration Research* (51) was the most productive journal, and *Stroke* (1346) was the most co-cited journal. An paper written by EZ Longa was the most influential reference, with the highest citation count. The hotspots and frontiers of this area of research were focused on the mechanisms of acupuncture, especially its neural regenerative or neuroprotective effects. This study used CiteSpace and VOSviewer for bibliometric analysis to provide researchers with information on the research status, hotspots, and trends in acupuncture for stroke research over the past 26 years.

## 1. Introduction

The risk of stroke has been increasing worldwide. Stroke is the leading cause of death and disability globally, imposing a significant economic burden.^[1–3]^ Stroke not only affects patients’ motor and cognitive functions but also has a strong association with the development of depression and even suicidal behavior.^[4–7]^ Stroke survivors are often left with complications, such as limb motor impairment, cognitive impairment, contractures, dysphagia, and urinary incontinence, which may require long-term rehabilitation, resulting in a reduced quality of life for the patients and a burden on society.^[8]^ Stroke has been recognized as a public health issue and has received extensive concern from medical practitioners.

Acupuncture is one of the most popular Chinese medical treatments and has been used as a preventive and therapeutic treatment for various diseases.^[9]^ As a safe treatment with few side effects, acupuncture is recommended as an effective strategy for stroke treatment and is increasingly gaining widespread acceptance.^[10–13]^ Acupuncture has been widely used for stroke rehabilitation and has shown definite efficacy for complications including limb movement dysfunction,^[14,15]^ cognitive impairment,^[16]^ swallowing disorders,^[17,18]^ insomnia,^[19]^ and depression.^[20]^ In addition, some researchers have found that acupuncture can prevent the occurrence of cerebral ischemia.^[21]^ To date, the field of acupuncture for stroke has received extensive attention from researchers; an increasing number of relevant studies are being carried out, and many related papers have been published. However, the research hotspots and directions in this field are still unclear.

Bibliometric analysis is a quantitative analysis of published academic literature using visualization tools, which can identify the research hotspots and frontiers in a specific discipline and help to predict the trends in its development. CiteSpace and VOSviewer, as the commonly used visualization applications, can help to draw co-occurrence network maps as well as citation network maps.^[22–24]^ With the help of these software programs, it is possible to carry out in-depth analysis of specific fields and provide researchers with reliable reference information. Two previous studies have conducted a bibliometric analysis on the literature published before 2012 in the field of acupuncture for stroke based on three databases: the Scopus database, the Web of Science, and the Clinical Trials Registry database.^[25,26]^ However, an increasing number of studies in this field have been performed in the past decade. It is necessary to conduct a bibliometric analysis of the existing published literature to reveal the research hotspots and trends and to identify the direction of future research in this area.

The purpose of this study is to explore the research status, hotspots, and trends on acupuncture for stroke worldwide.

## 2. Methods

### 2.1. Data source and search strategy

**Figure 1.**
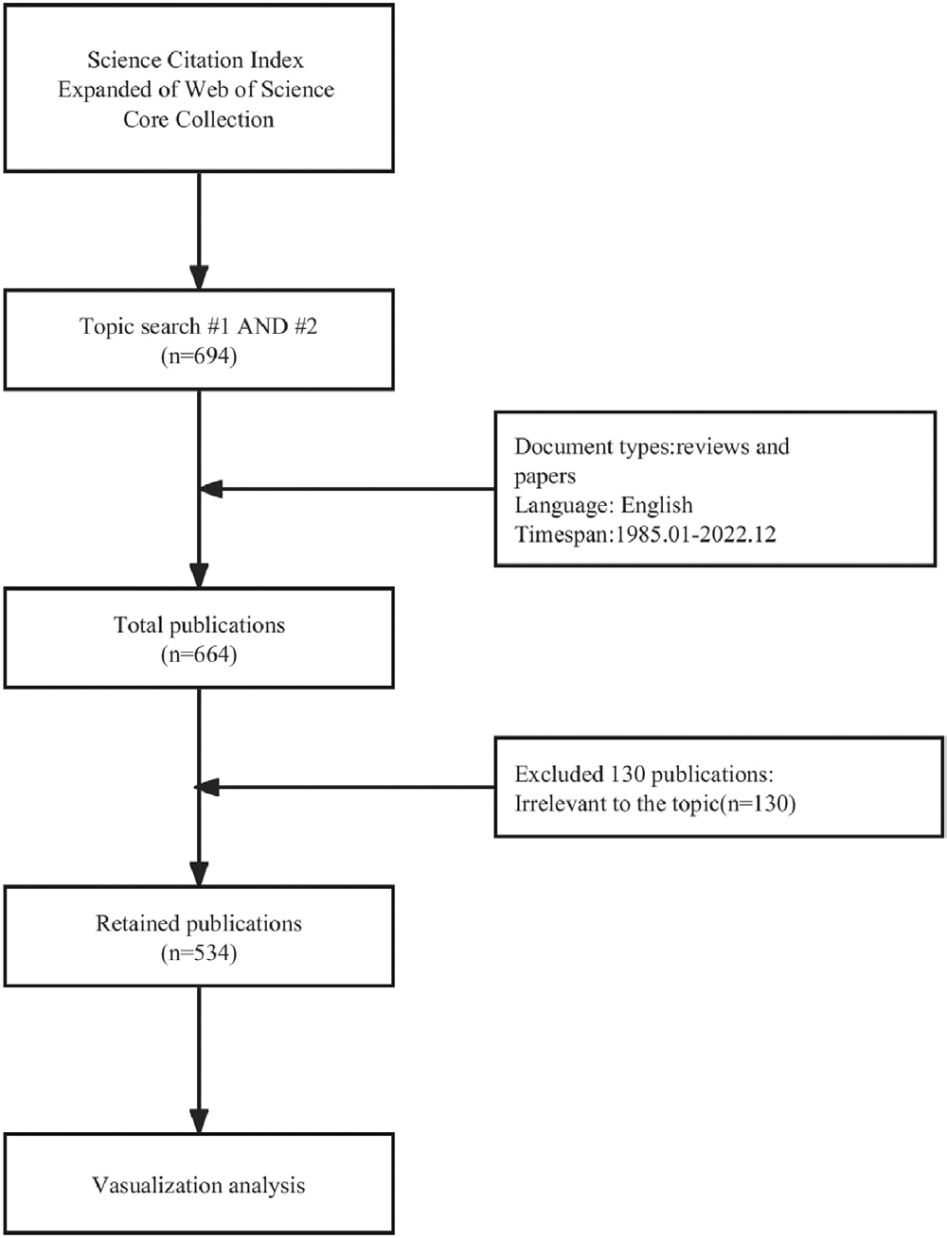
Flowchart of the process for study selection.

Data from the literature on acupuncture and stroke research were collected for review in this study. Table 1 shows the specific search strategy. Figure 1 shows the flowchart of study selection. According to the search criteria specified in Table 1, a total of 694 relevant papers were retrieved. After constraining the article type, language, and publication date, a total of 664 papers were obtained. Two independent researchers conducted a review of each paper’s title and abstract to further filter the papers. After excluding 130 papers that were deemed irrelevant to the topic, a final set of 534 papers was obtained.

**Table 1:** The topic search query (TS = Topic).

Set	Results	Search query
#1	10,616	TS = (Acupunture) OR TS = (Electroacupuncture)
		OR TS=(“electro-acupuncture”) OR TS = (Acupressure)
		OR TS = (Moxibustion) OR TS=(“AcupointInjection”)
		OR TS = (Acupoints) OR TS = (Pharmacoacupuncture)
		OR TS= (“Needle knife”) OR TS= (“catgut embedding)
		OR TS= (“catgut implantation at acupoint”)
		OR TS= (“embedding thread”)
#2	412,875	((TS = (stroke)) OR TS = (Cerebral hemorrhage)) OR
		TS = (Cerebralinfarction) OR TS = (apoplexy)
#3	694	#1 AND #2

### 2.2. Assessing

CiteSpace (6.2.R4) and VOSviewer (1.6.17) were used to conduct a bibliometric analysis of acupuncture for stroke. In this study, VOSviewer was used for visual analysis of countries, institutions, authors, journals, keywords, and references. CiteSpace was used to draw keyword burst maps and cited reference burst maps. The parameters of CiteSpace were set as follows: time slicing (1997–2022), years per slice (1), term source (all selections), node type (choose one at a time), and pruning (pathfinder).

## 3. Results

### 3.1. Publication output and time trends

**Figure 2.**
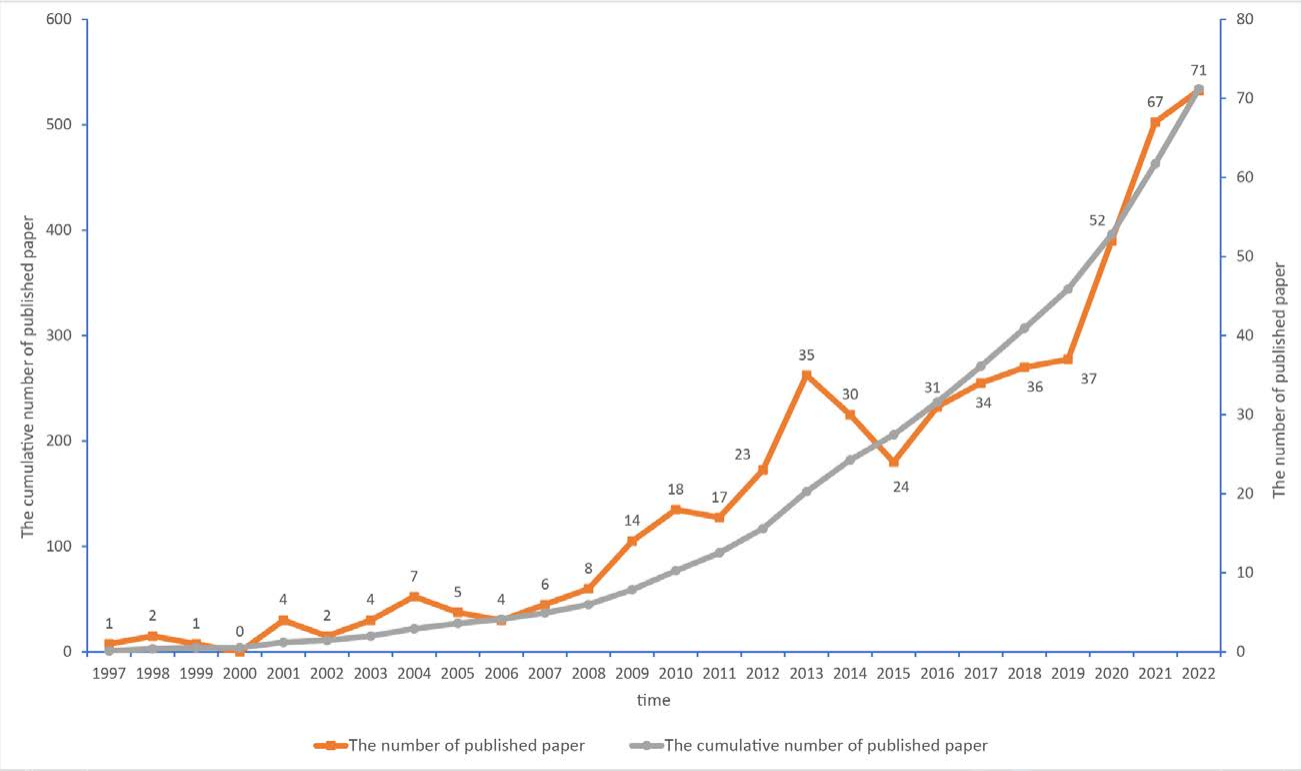
The number of publications per year and cumulative number of publications by SCI-E from 1997 to 2022 are described by a line chart, where gray represent the cumulative number of publications and yellow represent the number of publications per year. SCI-E = Science Citation Index-Expanded.

A total of 534 papers were obtained for visualization analysis, and the number of papers per year and cumulative number of papers are shown in Figure 2. From 1997 to 2008, there were no more than 10 research papers on acupuncture for stroke each year. Since 2009, the number of related research papers per year has increased, with a small peak in 2013 (35 publications, 6.55%). Thereafter, despite a brief, small decline, there was an overall trend of rapid growth in the number of relevant research papers per year, reaching a peak of 71 papers in 2022.

### 3.2. Analysis of countries

**Figure 3.**
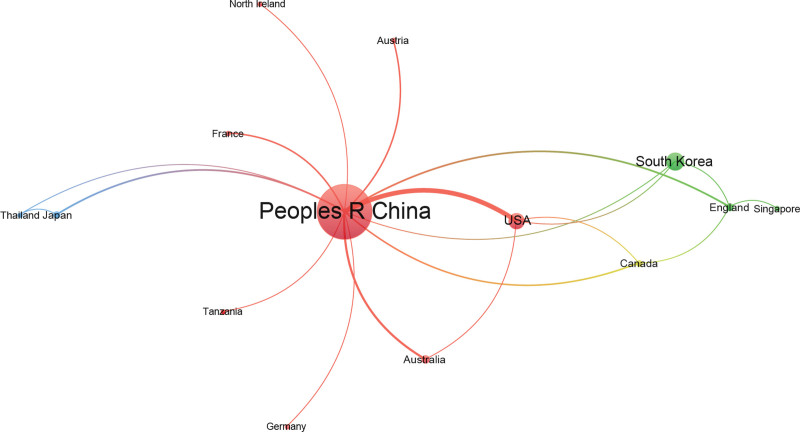
Map of active countries related to the research of acupuncture for stroke.

By knowing the number of papers in each country, one can quickly identify countries that have made outstanding contributions toward the treatment of stroke with acupuncture. Table 2 shows the top 5 countries with the most papers and their total and average citation frequencies. China was the leading country, with 452 papers (accounting for 84%), followed by South Korea (48 papers, 9%), the United States (38 papers, 7%), Australia (10 papers, 2%), and the England (8 papers, 1.5%). At present, research on acupuncture for stroke is mainly concentrated in China, South Korea, and the United States. The United States ranked first in the number of citations per paper (40.00). Although China had the largest number of papers published and the most citations in total, the average citation of Chinese papers was only 14.72, suggesting that the quality of papers needs to be improved. In all, 534 papers published in 24 different countries were included in the analysis (Fig. 3). Each node stands for a country. The lines between nodes correspond to the collaboration between countries, the wider the line, the greater the collaboration. The widest connecting line was between China and the United States, suggesting that these 2 countries have the strongest collaboration in the field of acupuncture for stroke.

**Table 2:** Top 5 countries and institutions related to the research of acupuncture for stroke.

Ranking	Country	Number of documents	Total citation	Average citation	Institution	Number of documents	Total citation	Average citation
1	Peoples R China	452	6655	14.72	Fujian Univ Tradit Chinese Med	43	1100	25.58
2	South Korea	48	1154	24.04	Guangzhou Univ Chinese Med	33	270	8.18
3	USA	38	1520	40.00	Tianjin Univ Tradit Chinese Med	32	331	10.34
4	Australia	10	107	10.70	Beijing Univ Chinese Med	25	191	7.64
5	England	8	318	39.75	China Med Univ	23	534	23.22

### 3.3. Analysis of institutions

**Figure 4.**
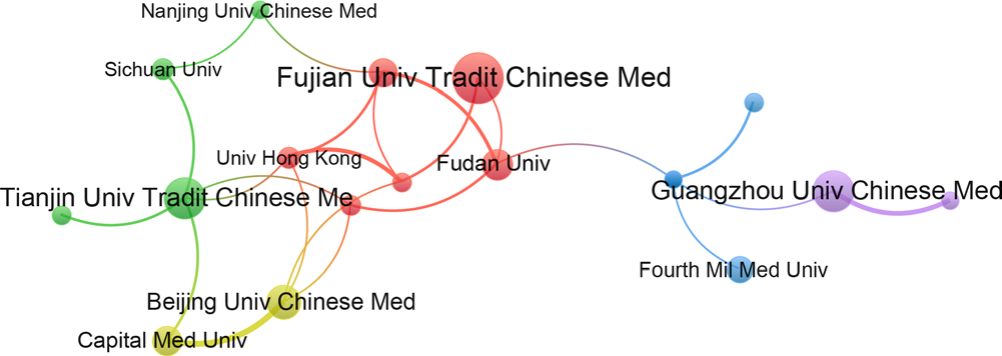
Map of active institutions related to the research of acupuncture for stroke.

The co-occurrence network of institutions can provide researchers with clear information on important institutions and potential collaborators in the research field. In total, 524 institutions have contributed to this field. Table 2 presents the top 5 institutions: Fujian University of Traditional Chinese Medicine (43), Guangzhou University of Traditional Chinese Medicine (33), Tianjin University of Traditional Chinese Medicine (32), Beijing University of Traditional Chinese Medicine (25), and China Medical University (23). These 5 institutions are all in China, indicating that China has an essential position in the field of acupuncture for stroke. The Fujian University of Traditional Chinese Medicine showed the highest average citation per paper. This shows that Fujian University of Traditional Chinese Medicine plays a crucial role in the field of acupuncture for stroke. For better visualization, a collaborative network map of 22 institutions with more than 10 publications was drawn using VOSviewer (Fig. 4). As shown in the figure, Beijing University of Traditional Chinese Medicine and Capital Medical University have the closest cooperation.

### 3.4. Analysis of authors and co-cited authors

In total, 2533 authors have contributed to the study of acupuncture for stroke. Table 3 shows the top 10 authors, who have a total of 183 publications, accounting for 34.26% of the total number of publications. The author with the highest number of publications is Tao Jing (34 publications), followed by Chen Lidian (28 publications), Liu Weilin (21 publications), Wang Qiang (21 publications), Huang Jia (20 publications), Xiong Lize (14 publications), Yang Shanli (12 publications), Lin Ruhui (11 publications), Choi Byung Tae (11 publications), and Shin Hwa Kyoung (11 publications).

**Table 3:** Top 10 authors and co-cited authors related to the research of acupuncture for stroke.

Rank	Author						Co-cited author	
	Name	Articles	Country	Total citation	Average citation	H-index	Name	Citation
1	Tao,Jing	34	China	1012	29.76	33	Longa, Ez	129
2	Chen, Li Dian	28	China	852	30.43	37	Wang, Q	107
3	Liu, Wei Lin	21	China	454	21.62	32	Li, J	65
4	Wang,Qiang	21	China	753	35.86	37	Tao, J	61
5	Huang,Jia	20	China	717	35.85	21	Wu, P	56
6	Xiong, Li Ze	14	China	614	43.86	39	Kim, Jh	52
7	Lin, Ru Hui	12	China	425	35.42	1	Wang, Y	52
8	Yang, Shan Li	11	China	284	25.82	20	Zhou, F	50
9	Choi, Byung Tae	11	South Korea	369	33.55	32	Liu, Y	50
10	Shin, Hwa Kyoung	11	South Korea	369	33.55	34	Zhang, sh	49

Among the top ten authors, Wang Qiang is from China Medical University, Xiong Lize is from Tongji University, Choi Byung Tae and Shin Hwa Kyoung are both from Pusan University, and the other 6 authors are from Fujian University of Traditional Chinese Medicine. This explains why China and the Fujian University of Traditional Chinese Medicine, respectively, are the most productive country and institution.

**Figure 5.**
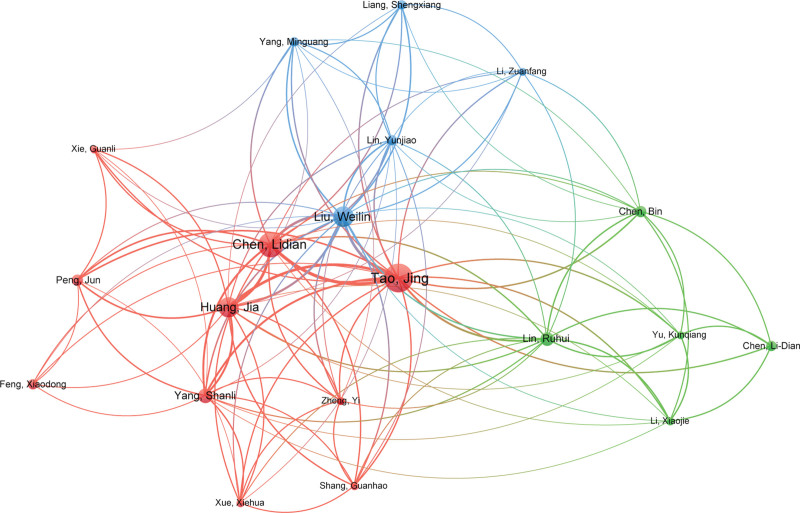
Map of active authors related to the research of acupuncture for stroke.

The co-author map helps identify the active and high-impact authors in the field. For better visualization, VOSviewer was used to create an author map based on up to 5 publications per author (Fig. 5). It can be seen from the figure that there is more cooperation and communication among authors from the same institution and less communication among authors from different institutions. In the future, closer cooperation and exchange between different institutions should be pursued.

Table 3 also shows the top ten cited authors of papers in the field of acupuncture for stroke. The most cited author is EZ Longa, from the University of California, USA, with 129 citations. The second most cited author is Wang Qiang, from the Air Force Medical University in China, with 107 citations. Regarding the rest of the top ten, Li Jun is from the University of Connecticut in France, Tao Jing is from the Fujian University of Traditional Chinese Medicine, Wu Ping is from the University of London in the United Kingdom, Kim Ji-hyun is from Pusan University in South Korea, Wang Yong is from the Tianjin University of Traditional Chinese Medicine, Zhou Fei is from the Shanghai Acupuncture Meridian Research Center, Liu Yi is from Capital Medical University, and Zhang Shihong is from Sichuan University.

### 3.5. Analysis of journals and co-cited journals

**Figure 6.**
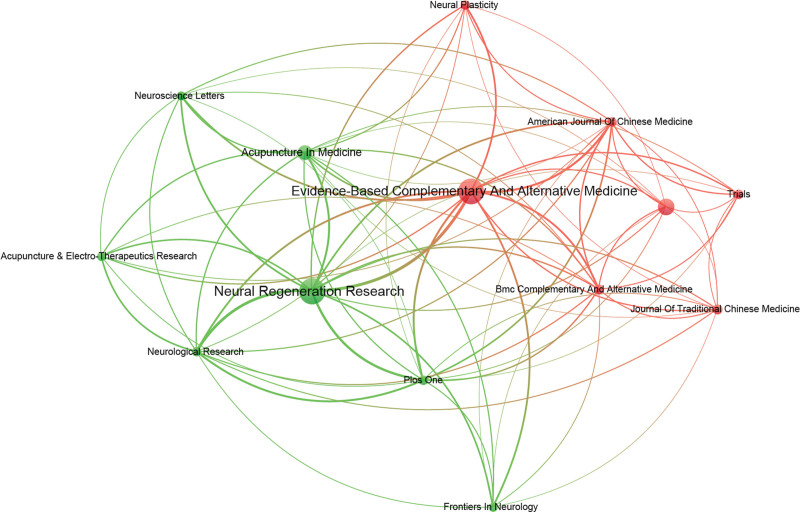
Map of active journals related to the research of acupuncture for stroke.

A total of 534 papers on acupuncture for stroke were published in 150 journals, of which 14 had more than 10 papers (Fig. 6). The top 10 scholarly journals that published the most papers on acupuncture for stroke research are shown in Table 4. The number of papers in these 10 journals accounted for 40.26% of the overall papers in the research field. Of these 10 journals, *Neural Regeneration Research* ranked first (51 papers, 9.55%), followed by *Evidence-Based Complementary and Alternative Medicine* (49 papers, 9.17%), *Medicine* (26 papers, 4.86%), *Acupuncture in Medicine* (21 papers, 3.93%), *BMC Complementary and Alternative Medicine* (12 papers, 2.24%), *Journal of Traditional Chinese Medicine* (12 papers, 2.24%), *Plos One* (11 papers, 2.05%), *American Journal of Chinese Medicine* (11 papers, 2.05%), *Neurological Research* (11 papers, 2.05%), and *Neural Plasticity* (11 papers, 2.05%). The journal with the highest impact factor was *Neural Regeneration Research*.

**Table 4:** Top 10 journals and co-cited journals related to the research of acupuncture for stroke.

Ranking	Journal	Number of documents	Citations	Average citations	IF	Co-cited journal	Citations	IF
1	*Neural Regeneration Research*	51	391	7.67	6.058	*Stroke*	1346	10.17
2	*Evidence-Based Complementary and Alternative Medicine*	49	512	10.45	2.65	*Evid-Based Compl Alt*	485	2.65
3	*Medicine*	26	41	1.58	1.817	*Zhongguo zhenjiu*	400	/
4	*Acupuncture in Medicine*	21	250	11.90	1.976	*Neurosci Lett*	381	/
5	*BMC Complementary and Alternative Medicine*	12	318	26.50	4.782	*Plos One*	325	3.752
6	*Journal of Traditional Chinese Medicine*	12	101	8.42	2.547	*Brain Res*	317	3.61
7	*Plos One*	11	294	26.73	3.752	*Am J Chinese Med*	293	6.005
8	*America Journal of Chinese Medicine*	11	337	30.64	6.005	*J Cerebr Blood Flow Met*	269	6.96
9	*Neurological Research*	11	287	26.09	2.529	*Zhen ci yan jiu*	265	/
10	*Neural Plasticity*	11	116	10.55	3.144	*J Altern Complem Med*	257	2.381

**Figure 7.**
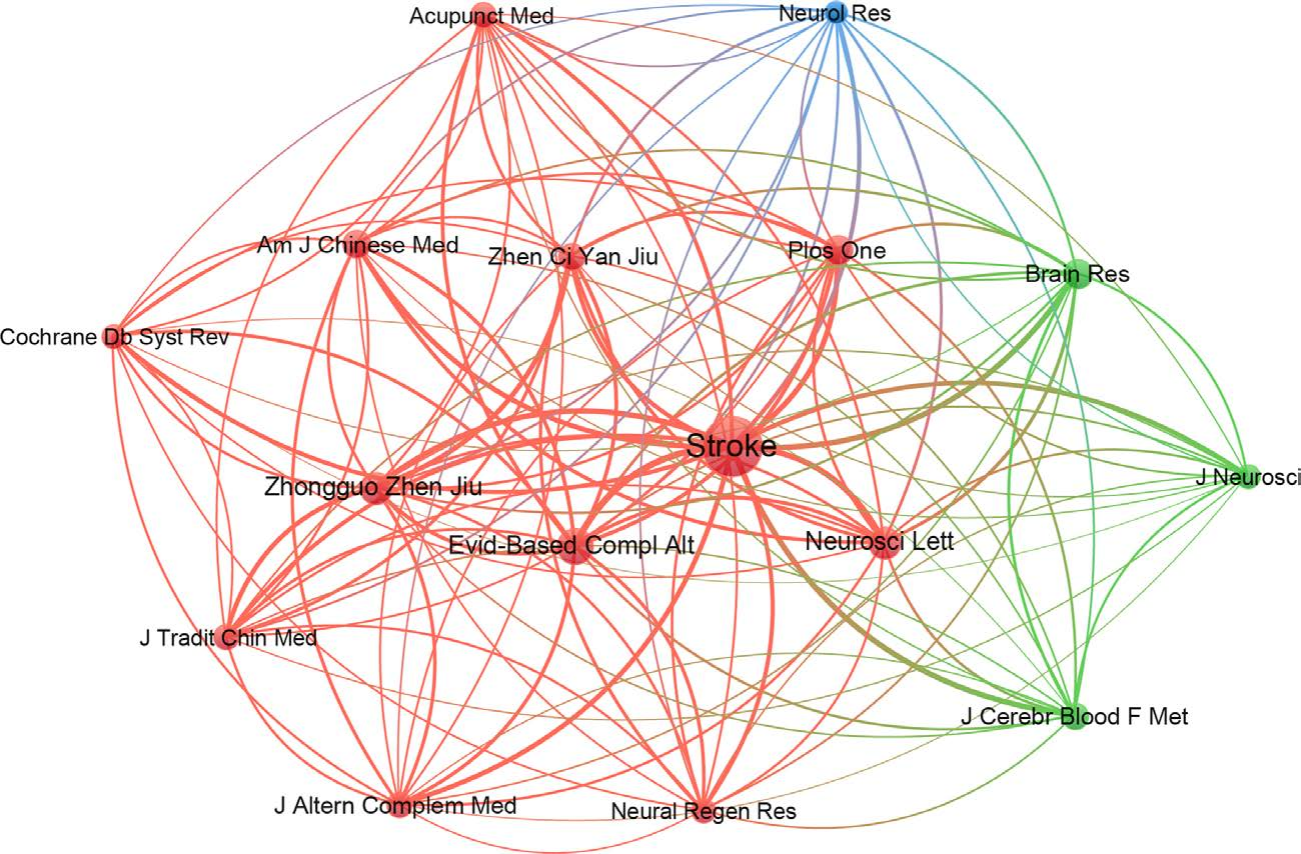
Map of cited journals related to the research of acupuncture for stroke.

There are a total of 4056 co-cited journals in the field of acupuncture for stroke, of which 16 journals have more than 200 co-cited papers (Fig. 7). The top ten co-cited journals in the field of acupuncture for stroke are also shown in Table 4. The top 3 are *Stroke, Evidence-Based Complementary Medicine*, and *Zhongguozhenjiu. Stroke* has the highest citation count, which means it is an important journal in the field and has made a significant contribution to the field.

### 3.6. Analysis of co-cited references

A total of 16,645 references were derived from 534 papers. The top 5 most co-cited references are shown in Table 5.

**Table 5:** Top 5 cited references related to the research of acupuncture for stroke.

Rank	Title	Citations	Year	Journal	First author
1	Reversible middle cerebral artery occlusion without craniectomy in rats	129	1989^[27]^	*Stroke*	Longa, E Z
2	Acupuncture in poststroke rehabilitation: a systematic review and meta-analysis of randomized trials	47	2010^[28]^	*Stroke*	Wu, Ping
3	Mechanisms of acupuncture therapy in ischemic stroke rehabilitation: a literature review of basic studies	42	2017^[29]^	*Int J Mol Sci*	Chavez, Lina M
4	Pretreatment with electroacupuncture induces rapid tolerance to focal cerebral ischemia through regulation of endocannabinoid system	37	2009^[30]^	*Stroke*	Wang, Qiang
5	Electroacupuncture ameliorates cognitive impairment through inhibition of NF-κB-mediated neuronal cell apoptosis in cerebral ischemia-reperfusion injured rats	36	2013^[31]^	*Mol Med Rep*	Feng, Xiaodong

The top paper, published by EZ Longa in *Stroke* in 1989, created a reliable and minimally invasive reversible ischemic stroke model in rats, which had been widely used to simulate human cerebrovascular diseases in experimental studies of permanent and transient regional cerebral ischemia.^[27]^

The second-ranking paper is a review published by Wu et al in 2010 in *Stroke*, which showed convincing evidence supporting a role for acupuncture in poststroke rehabilitation.^[28]^

The third-ranking paper is a review by Chavez Lina M et al, published in the *International Journal of Molecular Sciences* in 2017. This review included 40 papers summarizing the mechanisms of acupuncture and electroacupuncture (EA) for the rehabilitation of ischemic stroke and summarized the acupoints commonly used in published studies.^[29]^

The fourth-ranking paper was published by Wang Qiang et al in *Stroke* in 2009, reporting that EA pretreatment could have an early protective effect on transient cerebral ischemia by increasing endocannabinoids and 2-arachidonoylglycerol.^[30]^

The fifth-ranking paper, published by Feng Xiaodong et al in 2012, evaluated the therapeutic effect of EA on poststroke cognitive impairment and explored the potential molecular mechanism. They found that EA had therapeutic effects on poststroke cognitive impairment. The mechanism may consist of the inhibition of NF-KB-mediated apoptosis of nerve cells.^[31]^

### 3.7. Analysis of references with the strongest citation bursts

**Figure 8.**
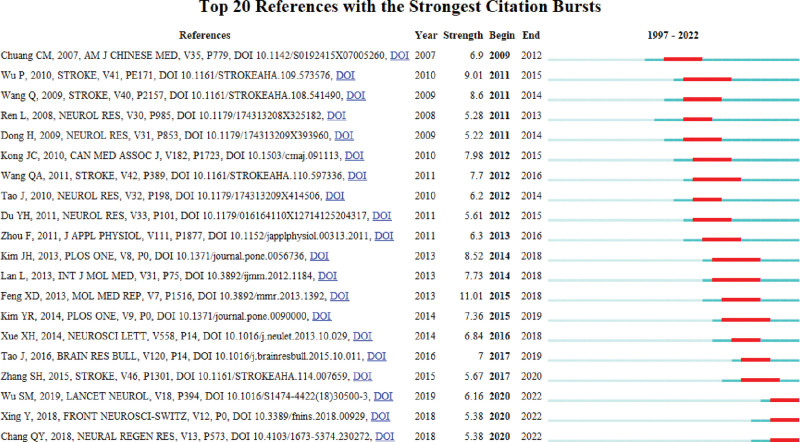
Top 20 references with the strongest citation bursts.

A citation burst indicates a reference that has been cited heavily by papers in a period. The top 20 references with the strongest citation bursts in the field of acupuncture for stroke are presented in Figure 8, reflecting the dynamics of this area of research. The red line segments indicate the times of the citation bursts. The earliest burst of references began in 2007. Four papers were published in *Stroke* (20%) and five papers in *Neural Regeneration Research* (25%). More than half of the 20 papers were published in influential journals with impact factors >5, which may be the reason why these papers are relatively popular. There were 3 references with citation bursts from 2018 to 2022, published by Wu Simiao (2019), Xing Ying (2018), and Qwang-Yuen Chang (2018). Of these, the paper by Wu had the strongest burst.

### 3.8. Analysis of co-occurring keywords

The keywords of an paper summarize its key content, and those with high frequency can be indicative of research hotspots in the research field. Table 6 shows the top 10 keywords with the highest frequency and centrality. The most frequent keyword is “EA” (256 times), followed by “Stroke” (249 times), “Acupuncture” (232 times), “Expression” (80 times), “Stimulation” (76 times), “Brain” (74 times), “Recovery” (68 times), “Rehabilitation” (66 times), “Activation” (65 times), and “Arterial occlusion” (64 times). As an indicator to measure the importance of keywords, the value of centrality has a positive correlation with the importance of keywords. That is, the higher the value of the centrality of the keyword, the greater the influence, and the more important its position. The top 5 keywords by centrality are “Brain” (0.3), “Cerebral ischemia” (0.19), “Stroke” (0.18), “EA” (0.17), and “Acupuncture” (0.16). Activation, arterial occlusion, stimulation, expression, and recovery also have high centrality.

**Table 6:** The top 10 keywords with the highest frequency and centrality.

Ranking	Frequencies	Keyword	Centrality	Keyword
1	256	Electroacupuncture	0.3	Brain
2	249	Stroke	0.19	Cerebral ischemia
3	232	Acupuncture	0.18	Stroke
4	80	Expression	0.17	Electroacupuncture
5	76	Stimulation	0.16	Acupuncture
6	75	Brain	0.13	Activation
7	68	Recovery	0.12	Artery occlusion
8	66	Rehabilitation	0.12	Stimulation
9	65	Activation	0.11	Expression
10	64	Artery occlusion	0.1	Recovery

**Figure 9.**
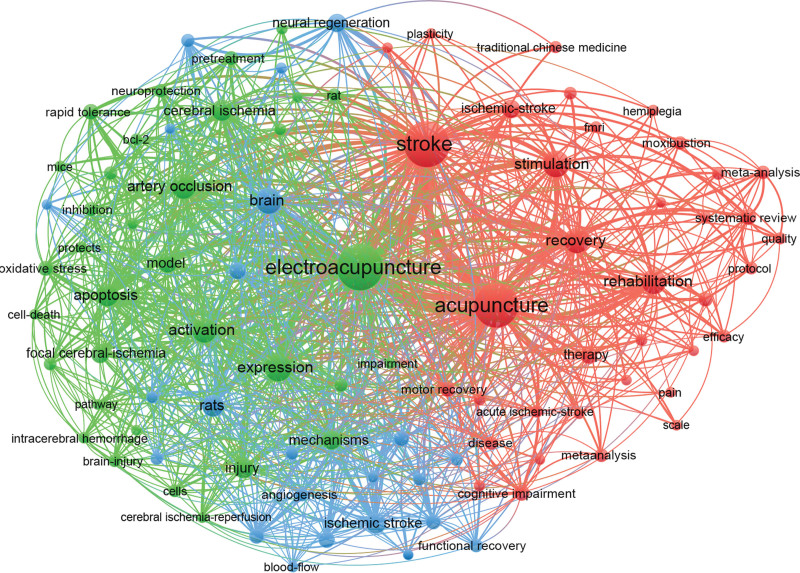
Map of co-occurring keywords related to the research of acupuncture for stroke.

VOSviewer was used to visualize the co-occurrence of keywords in publications on acupuncture for stroke (Fig. 9). For better visualization, only 94 keywords that occur more than 10 times are shown in the figure, which resulted in the identification of 3 clusters, shown in different colors. Cluster 1 (red) refers to stroke complications and the burden of disease, with the main keywords being cognitive impairment, hemiplegia, pain, and quality of life. Cluster 2 (green) refers to the mechanism of acupuncture, and the main keywords are apoptosis, mechanism, hippocampus, neuroprotection, and oxidative stress. Cluster 3 (blue) refers to rehabilitation, and the main keywords are functional recovery, neural regeneration, and synaptic plasticity.

### 3.9. Analysis of keywords with the strongest citation bursts

**Figure 10.**
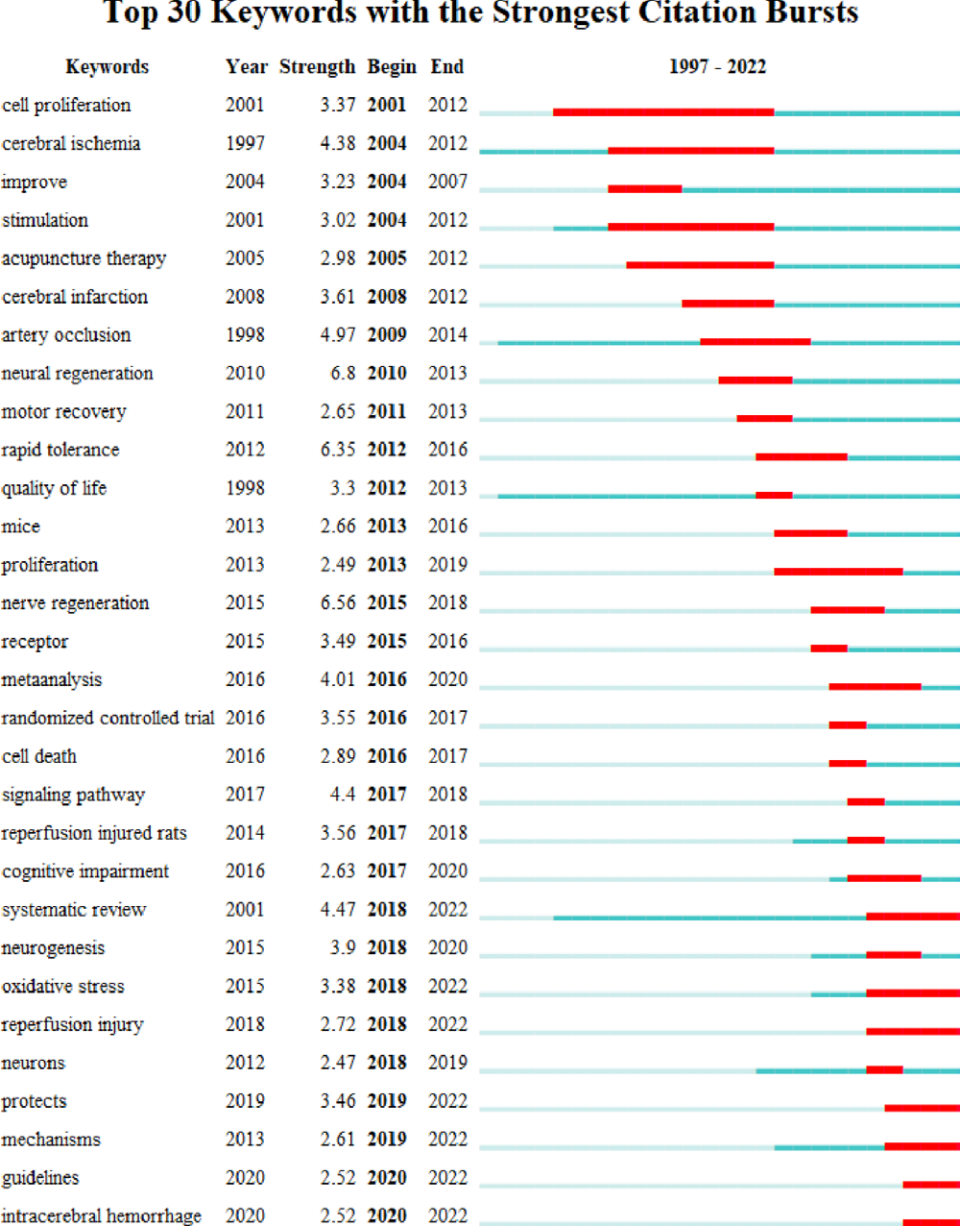
Top 30 keywords with the strongest citation bursts.

An analysis of burst keywords can extract keywords with a high frequency change rate and a fast growth rate from the included literature to reflect the changing hotspots and predict the future development trend. Burst intensity is an important predictor of burst detection. We identified a total of 30 burst keywords using CiteSpace (Fig. 10). The red line segments indicate the times of keyword bursts, and the intensity indicates the level of impact. The keywords with the highest intensity are neural regeneration, rapid tolerance, and artery occlusion. The 7 keywords that appeared most recently are systematic review, oxidative stress, reperfusion injury, protects, mechanisms, guidelines, and intracerebral hemorrhage.

## 4. Discussion

### 4.1. General information

The number of papers published on acupuncture for stroke was below 10 per year until 2008, indicating that this research field had not received considerable attention. Since 2009, the number of papers published each year has steadily increased. Since 2019, there have been 2 consecutive years in which the number of papers in publication increased by more than 10, namely, 2019 to 2020 (15 papers) and 2020 to 2021 (15 papers), demonstrating that this research field is booming and has received increasing attention. The analysis of countries shows that the country with the greatest numbers of papers and citations is China, which may be due to the Chinese origins of acupuncture. However, the United States has the highest number of citations per paper (40), indicating that the quality of these research papers is widely recognized to be high. As shown in the figure, in terms of national cooperation, China and the United States have the closest connection, while there are fewer links between other countries; a stronger network of collaboration should be established among more countries.

By our analysis of institutions and authors, Fujian University of Traditional Chinese Medicine has the most publications and the highest average citation count, and the most prolific author is also from Fujian University of Traditional Chinese Medicine, illustrating that this institution and its researchers have an important position in the field of acupuncture for stroke and have made important contributions. Tao Jing, the most prolific author in the field of acupuncture for stroke research, leads a research team focusing on the mechanism of EA for ischemic stroke. They found that EA alleviated neurological deficits by stimulating the proliferation and differentiation of nerve stem cells through activating the Notch signaling pathway.^[32,33]^ In addition, EA at QuChi and ZuSanLi can exert neuroprotective effects on rats with ischemia-reperfusion injury by inhibiting the TLR4/NF-κB pathway and activating the PI3K/Akt pathway.^[34,35]^ A recent study from the team showed that EA promotes endogenous neural stem cell differentiation through exosomal microRNA 146b to improve neurological damage after ischemic stroke.^[36]^ These studies provide evidence revealing the mechanism of acupuncture in the treatment of stroke.

In terms of journal analysis, *Neural Regeneration Research* and *Evidence-Based Complementary Medicine* were the 2 most productive journals, with impact factors of 6.058 and 2.650, respectively. However, the most frequently cited journal was *Stroke*, with an impact factor of 10.17. Among the top 10 journals in terms of the number of articles published and the frequency of citations, only 5 journals (25%) had an impact factor >5, indicating that it is still a challenge for researchers to publish papers on acupuncture for stroke in high-impact journals; accordingly, researchers must enhance the quality of their papers. As significant and highly cited journals, these journals showcase papers on vital research findings in this field. Future researchers should keep an eye on these journals for quick access to the latest international information and research progress in the field of acupuncture for stroke.

### 4.2. Research hotspots

Keywords can reflect the core theme of the paper. By combining information on highly co-cited authors and highly cited references to identify the hotspots of research on acupuncture for stroke, researchers can explore the distribution of topics in this research area. Keyword co-occurrence analysis shows that the 3 most frequent keywords are EA, stroke, and acupuncture.

Electroacupuncture is commonly used in combination with traditional stroke rehabilitation. Clinical studies have demonstrated that EA can effectively treat various sequelae of stroke, including dysphagia,^[37]^ pain,^[38]^ aphasia,^[39]^ and urinary incontinence.^[40]^ A review of animal experiments suggested that EA could alleviate ischemic brain injury by engaging in various signaling pathways and regulating multiple molecular processes such as cell apoptosis, inflammation, and autophagy.^[41]^ Another study found that EA after ischemic brain injury might achieve neuroregeneration or neuroprotection by inhibiting apoptosis, reducing glutamate excitotoxicity, enhancing cerebral blood flow and growth factor production, modulating oxidative damage, maintaining blood-brain barrier integrity, and increasing cerebral ischemic tolerance.^[42]^ In addition to EA, commonly employed clinical treatment methods include manual acupuncture, acupressure at specific acupuncture points, and scalp acupuncture. Studies had found that EA generated greater activation in the somatosensory cortex, while manual acupuncture primarily results in deactivation of limbic system structures.^[43–45]^ Acupressure can enhance the flow of energy (qi) by stimulating meridians.^[46]^ Clinical studies suggested that scalp acupuncture improved neurological function deficits and promoted the recovery of motor functions in stroke patients by increasing cerebral blood flow and oxygen supply.^[47]^ In terms of the choice of acupoints, the most commonly used acupuncture points are mainly in the limbs, and the most frequently used acupoints are Yanglingquan and Waiguan.^[48]^

The keyword cluster analysis map drawn by VOSviewer shows that the existing researches on acupuncture for stroke have 3 main aspects, namely, stroke complications and the burden of disease, the mechanism of acupuncture, and rehabilitation. Among the many complications of stroke, hemiplegia, cognitive impairment, and pain are the 3 most concerning aspects.

Hemiplegia, also known as unilateral motor disorders following stroke, accounts for 50% to 83% of all complications.^[49–51]^ Hemiplegia has a disability rate of up to 80%, seriously affecting the patient’s activities of daily living, reducing the patient’s quality of life, and placing immense pressure on the family and society.^[52]^ Studies have confirmed that acupuncture has satisfactory immediate and long-term effects on patients with hemiplegia after stroke, and early acupuncture intervention may enhance the rehabilitation of hemiplegic patients.^[53,54]^ Increasingly many countries and regions recommend acupuncture as an important treatment for stroke rehabilitation.^[55]^ However, the underlying mechanism of acupuncture in the treatment of poststroke motor dysfunction is still unclear and has become a research hotspot in recent years.

Poststroke cognitive impairment (PSCI) is one of the major complications occurring after stroke. Previous studies have evaluated the prevalence of PSCI and the factors influencing it. The latest study^[56]^ included 24,055 patients with first-time ischemic stroke and found a PSCI prevalence of 78.7% in this population. The incidence of PSCI^[57,58]^ is related not only to age, education level, and region but also to features of the stroke itself, such as infarct volume and stroke location. Stroke patients can have different degrees of PSCI, ranging from mild to severe. As an effective, safe, and affordable nonpharmacological treatment, acupuncture is used as an alternative supportive therapy for the treatment of PSCI. Multiple systematic reviews and meta-analyses of randomized controlled trials^[16,59,60]^ have shown that acupuncture can effectively promote the rehabilitation of patients with cognitive impairment after cerebral infarction and improve their scores on the Mini-Mental State Examination, the Montreal Cognitive Assessment, and Hasegawa Dementia Scale. In a systematic review^[61]^ of 22 studies (with a total of 647 patients) that used neuroimaging to investigate the therapeutic mechanisms of acupuncture for mild cognitive impairment, researchers found that the cingulate cortex, hippocampus, and prefrontal cortex were the 3 brain regions most influenced by acupuncture. However, the correlation between brain responses and clinical outcomes remains to be investigated. There is still a lack of high-quality randomized controlled trials published in the field of acupuncture for cognitive impairment. More attention could be given to this area in the future by researchers specializing in relevant fields.^[62]^

Pain is an unbearable symptom that affects physical, emotional, and cognitive functioning, leading to a lower quality of life and a higher risk of death. A variety of age groups, including adolescents and elderly people, suffer from pain. A recent large-scale national cross-sectional study^[63]^ showed that 60.02% of middle-aged and elderly people in China had experienced body pain. Another study, which included data from 42 countries and territories, showed that 23.6% of adolescents reported experiencing localized specific single-site pain.^[64]^ Chronic pain, due to its complex pathophysiology, is often combined with psychiatric disorders such as anxiety, depression, and insomnia, leading to ineffective drug treatment.^[65,66]^ A variety of age groups, including adolescents and elderly people, suffer from pain. Studies have shown that analgesic use is often accompanied by many side effects, such as nausea and vomiting, constipation, cognitive impairment, and respiratory depression, which adversely affect the quality of life of patients.^[67,68]^ Acupuncture has been chosen as a pain management option because of its safety and lack of side effects compared to analgesics. Acupuncture treatment helps reduce the use of analgesics for pain patients, and the long-term effects of acupuncture are satisfactory.^[69,70]^

In terms of co-cited authors, EZ Longa, a scholar from the University of California in the United States, has the top citation. In his paper published in *Stroke* in 1989,^[27]^ he created a reliable and minimally invasive rat model of reversible ischemic stroke, which had been widely used in experimental studies of permanent and transient regional cerebral ischemia. Wang Qiang from the Air Force Medical University of China is the second most highly cited author. He focused on animal studies of EA for ischemic stroke, its application in anesthesia, and its mechanism of action. The team found that EA preconditioning provided potent protection against transient ischemic injury by inhibiting the release of HMGB1 through activation of α7nAChR.^[30,71,72]^ Furthermore, a review^[73]^ conducted by the team summarized the important role of acupuncture in the perioperative period. Acupuncture could not only reduce the consumption of anesthetics but also reduce perioperative complications. At the same time, acupuncture helped to regulate body balance, improve organ function, and keep the body in equilibrium during the perioperative period. These findings indicated that previous studies on acupuncture for stroke mostly focused on animal experiments, and researchers were committed to exploring the mechanism of action of acupuncture.

According to the 5 most cited references, we found that animal experiments and clinical trials in the field of acupuncture for stroke researches were being actively performed by researchers from various countries. In general, animal experiments are more favored by scholars. In the future, additional high-quality, large-sample clinical studies are expected to be conducted collaboratively among researchers in various countries and institutions.

### 4.3. Research frontiers

In accordance with the analysis of references with the strongest citation bursts, 2 of the top 3 recently cited references^[41,42]^ discussed the neural regenerative or neuroprotective effects of acupuncture/EA for ischemic stroke.

According to the keyword burst map drawn by CiteSpace, among the most recent keywords, the top four in burst strength are “systematic review,” “oxidative stress,” “protects,” and “mechanisms.” Research on the mechanisms of acupuncture for stroke is becoming the frontier of the field. Compared with hemorrhagic stroke, research on the mechanism of acupuncture for ischemic stroke has attracted more attention from scholars, which may be related to the higher incidence of ischemic stroke.^[74]^ Many basic research studies have explored the mechanism of acupuncture, and the results show that acupuncture can effectively promote the rehabilitation of ischemic stroke and reduce the infarct volume and neurological deficit after stroke.^[29]^ Furthermore, a review^[75]^ summarized the possible mechanisms of action involved in the current treatment of ischemic stroke with acupuncture: (1) promoting nerve regeneration and cell proliferation, acupuncture can promote the proliferation, migration, and differentiation of neural stem cells to reduce neurological deficits^[36,76]^; (2) alleviating neuroinflammation, EA reduces motor injury by inhibiting neuroinflammation mediated by microglia in the peri-infarct sensorimotor cortex^[77]^; (3) inhibition of neuronal apoptosis, EA can inhibit neuronal apoptosis in a variety of ways, reduce neurological deficits, and restore damaged brain cells^[78–80]^; (4) regulation of oxidative stress, several studies have shown that acupuncture alleviates ischemia-induced oxidative stress by modulating a series of molecular signaling pathways involved in redox regulation.^[81–83]^

In conclusion, the research trends on acupuncture for stroke mainly highlight its mechanism of action, especially its neural regenerative or neuroprotective effects.

## 5. Limitations

Some limitations of this study still need to be addressed. First, our study is limited papers published up to December 31, 2022, and we may have overlooked some of the most recently published papers. Second, our study restrict the language of the papers to English, which may have led to language bias. In addition, this study includes only papers indexed in the Web of Science Core Collection database; some papers not included in this database are missed. Finally, the current paper lacks the ability to compare the clinical efficacy of various acupuncture methods and lacks a connection with clinical outcomes.

## 6. Conclusion

In conclusion, this study used CiteSpace and VOSviewer for bibliometric analysis to inform researchers about the status, hotspots, and trends on acupuncture for stroke research over the past 26 years. The current status of research indicates that publications in the field of acupuncture for stroke are growing rapidly and the field is receiving extensive attention. China has the largest number of papers in publication. The closer cooperation and exchange between different institutions and countries should be pursued. Researchers should strive to improve the quality of their papers, increase the number of papers published in high-impact journals, and improve the influence of their research field. Hemiplegia, cognitive impairment, and pain are the 3 stroke complications that have received the most attention. The research hotspots and trends on acupuncture for stroke involve exploring the mechanisms of action of acupuncture, especially its neural regenerative and neuroprotective effects.

## Author contributions

**Funding acquisition:** Hai-Bo Yu.

**Project administration:** Hai-Bo Yu.

**Supervision:** Hai-Bo Yu.

**Visualization:** Chang-Jiang Cheng.

**Writing – original draft:** Chang-Jiang Cheng.
